# Supplementary material for: Hepatitis B vaccination status and knowledge, attitude, and practice regarding Hepatitis B among preclinical medical students of a medical college in Nepal
Source: PLoS One. 2020 Nov 23;15(11):e0242658. doi: 10.1371/journal.pone.0242658 (PMC7682811; doi:10.1371/journal.pone.0242658)
Supplement: S1 File — (DOCX) [file pone.0242658.s001.docx]

**Online e-supplement**

**Hepatitis B vaccination status and knowledge, attitude, and practice regarding Hepatitis B among preclinical medical students of a medical college in Nepal**

Dhan Bahadur Shrestha^1*^, Manita Khadka^2^, Manoj Khadka^2^, Prarthana Subedi^2^, Subashchandra Pokharel^2^, Bikash Bikram Thapa^2^

^1^Mangalbare Hospital, Morang, Nepal

^2^Nepalese Army Institute of Health Sciences (NAIHS), Shree Birendra Hospital, Chhauni, Kathmandu, Nepal

**Questionnaire**

**Informed Consent**

This study aims to assess the knowledge, attitude, and practice towards Hepatitis B and vaccination status of preclinical medical students of a medical college in Kathmandu valley. Your participation in the study is voluntary. It will take less than 5 minutes to fill the form. The responses will be kept confidential and used for study purpose only. So, we expect sincere answers from you as the responses might help to plan and implement steps to improve the hepatitis B vaccination status in near future. Hoping for your support & coordination.

I have read the above information and voluntarily agree to participate in the study.

1. **General information**

**Gender:** Male Female

**Age (in completed years): ..........**

**Academic year:** 1^st^ year, MBBS 2^nd^ year, MBBS

| **Statement** | **Strongly disagree** | **Disagree** | **Neutral** | **Agree** | **Strongly agree** |
| --- | --- | --- | --- | --- | --- |
| Hepatitis B is caused by a virus. |  |  |  |  |  |
| Hepatitis B can be transmitted by |  |  |  |  |  |
| 1. Infected mother to fetus |  |  |  |  |  |
| 1. Contaminated blood & body fluids |  |  |  |  |  |
| 1. Unprotected sex with infected ones |  |  |  |  |  |
| 1. Casual contact (shaking hands) |  |  |  |  |  |
| 1. Unsterilized syringes/needles |  |  |  |  |  |
| 1. Coughing/sneezing |  |  |  |  |  |
| 1. Contaminated food/water |  |  |  |  |  |
| Hepatitis B can cause liver cancer. |  |  |  |  |  |
| Health care workers are at increased risk of getting Hepatitis B than general population. |  |  |  |  |  |
| Hepatitis B can be prevented by |  |  |  |  |  |
| 1. Vaccination |  |  |  |  |  |
| 1. Antivirals |  |  |  |  |  |
| 1. Avoiding sharp needle/syringe injury |  |  |  |  |  |
| 1. Avoiding contaminated water/food |  |  |  |  |  |
| 1. Using gloves when handling body fluid |  |  |  |  |  |

1. **Knowledge related to Hepatitis B** (Please select the most appropriate option)
2. **Attitude related to hepatitis B** (Please select the most appropriate option)

| **Statement** | **Strongly disagree** | **Disagree** | **Neutral** | **Agree** | **Strongly agree** |
| --- | --- | --- | --- | --- | --- |
| I feel uncomfortable sitting with Hepatitis B infected person. |  |  |  |  |  |
| I don’t mind shaking hands/ hugging with Hepatitis B infected person. |  |  |  |  |  |
| I believe Hepatitis B vaccine is safe and effective. |  |  |  |  |  |
| I believe health care workers should receive Hepatitis B vaccination. |  |  |  |  |  |
| I don’t need Hepatitis B vaccination because I am not at risk. |  |  |  |  |  |

1. **Practice related to hepatitis B** (Please select the most appropriate option)

| **Statement** | **Strongly disagree** | **Disagree** | **Neutral** | **Agree** | **Strongly agree** |
| --- | --- | --- | --- | --- | --- |
| I ask/use a new blade for shaving/hair cutting. |  |  |  |  |  |
| I ask for a new syringe before injection. |  |  |  |  |  |
| I ask for sterilized equipment for ear/nose piercings. |  |  |  |  |  |
| I always report for needle pricks / sharp injuries. |  |  |  |  |  |
| I attend hepatitis B related awareness program |  |  |  |  |  |

1. **Hepatitis B vaccination status**
2. Have you been vaccinated against Hepatitis B?

(*Hepatitis B vaccine was introduced after 2002 AD in Nepal)

- Yes
- No

1. How many doses of Hepatitis B vaccine did you receive? (*Only if vaccinated)

- One dose
- Two doses
- Three doses
- More than three doses

1. What are the reasons for you not being vaccinated? (*If not vaccinated)

- Low risk of Hepatitis B
- No vaccination program offered
- High vaccination fees
- Others (Please specify)..........
