# Supplementary material for: Hepatitis B vaccination status and knowledge, attitude, and practice regarding Hepatitis B among preclinical medical students of a medical college in Nepal
Source: PLoS One. 2020 Nov 23;15(11):e0242658. doi: 10.1371/journal.pone.0242658 (PMC7682811; doi:10.1371/journal.pone.0242658)
Supplement: Attachment — Submitted filename: Shrestha PONE-D-20-25582 Hamm suggested changes.pdf [file pone.0242658.s003.pdf]

PLOS ONE

Hepatitis B vaccination status and knowledge, attitude, and practice regarding Hepatitis B among preclinical medical students of a medical college in Nepal

--Manuscript Draft--

**Manuscript Number:**	PONE-D-20-25582
**Article Type:**	Research Article
**Full Title:**	Hepatitis B vaccination status and knowledge, attitude, and practice regarding Hepatitis B among preclinical medical students of a medical college in Nepal
**Short Title:**	Knowledge, attitude, and practice regarding Hepatitis B among medical students
**Corresponding Author:**	Dhan Bahadur Shrestha, MBBS<br>Mangalbare Hospital<br>Urlabari, 1 NEPAL
**Keywords:**	Attitude; Hepatitis B; Knowledge; Medical students; Nepal; Practice; Vaccination
**Abstract:**	Background<br><br>Hepatitis B imposed a major public health problem with an increased risk of occupational exposure among health care workers. Medical students are also at increased risk due to a lack of mandatory vaccination programs and accidental needle stick injuries during clinical years. This demands the need for Hepatitis B vaccination of medical students before they enter clinical years. This study was conducted to determine the Hepatitis B vaccination status along with the knowledge, attitude, and practice regarding Hepatitis B among preclinical medical students of a medical college in Nepal.<br><br>Materials and Methods<br><br>This descriptive cross-sectional study was conducted among first and second-year medical students of a medical college in Kathmandu, Nepal from 6 th July to 14 th July 2020. The whole sampling technique was used. Data were collected using a pretested, self-administered questionnaire which was mailed to individuals and analyzed in the statistical package for social sciences version-22.<br><br>Results<br><br>A total of 181 students participated in the study out of 198, giving a response rate of 91.4%. Among the study participants, only 67 (37%) were fully vaccinated against Hepatitis B while 71 (39.2%) were never vaccinated. For the majority (74.6%) of the non-vaccinated participants, the main reason for not getting vaccination was a lack of vaccination programs. Regarding the knowledge, attitude, and practice, half the study participants (n=92, 50.8%) had good knowledge, attitude and practice regarding hepatitis B. The median knowledge, attitude and practice score towards Hepatitis B were 61.00 (57.00-66.00), 20(18.00- 21.00) and 21(19.00- 23.00) respectively.<br><br>Conclusions<br><br>The majority of preclinical medical students were not fully vaccinated against Hepatitis B and only half of them, having acceptable knowledge, attitude and practice towards hepatitis B, which makes them vulnerable to hepatitis B during clinical years. This might represent the scenario of not only Nepal but South Asian countries and creates an alarm to taking the vaccination programs seriously.Since the majority of the non-vaccinated participants responded unavailability of vaccination program as the main cause, we strongly recommend the provision of the Hepatitis B vaccination program to the pre-clinical medical students so that they will be protected when they enter clinical training.
**Order of Authors:**	Dhan Bahadur Shrestha, MBBS
	Manita Khadka

Powered by Editorial Manager® and ProduXion Manager® from Aries Systems Corporation

	Manoj Khadka
	Prarthana Subedi
	SubashChandra Pokharel
	Bikash Bikram Thapa
**Additional Information:**	
**Question**	**Response**
**Financial Disclosure**<br><br>Enter a financial disclosure statement that describes the sources of funding for the work included in this submission. Review the submission guidelines for detailed requirements. View published research articles from *PLOS ONE* for specific examples.<br><br>This statement is required for submission and **will appear in the published article** if the submission is accepted. Please make sure it is accurate.<br><br>**Unfunded studies**<br>Enter: *The author(s) received no specific funding for this work.*<br><br>**Funded studies**<br>Enter a statement with the following details:<br>• Initials of the authors who received each award<br>• Grant numbers awarded to each author<br>• The full name of each funder<br>• URL of each funder website<br>• Did the sponsors or funders play any role in the study design, data collection and analysis, decision to publish, or preparation of the manuscript?<br>• **NO** - Include this sentence at the end of your statement: *The funders had no role in study design, data collection and analysis, decision to publish, or preparation of the manuscript.*<br>• **YES** - Specify the role(s) played.<br><br>* typeset	The author(s) received no specific funding for this work.
**Competing Interests**<br><br>Use the instructions below to enter a competing interest statement for this submission. On behalf of all authors, disclose any competing interests that	The authors have declared that no competing interests exist.

could be perceived to bias this work—acknowledging all financial support and any other relevant financial or non-financial competing interests.<br><br>This statement **will appear in the published article** if the submission is accepted. Please make sure it is accurate. View published research articles from *PLOS ONE* for specific examples.<br><br>**NO authors have competing interests**<br><br>Enter: *The authors have declared that no competing interests exist.*<br><br>**Authors with competing interests**<br><br>Enter competing interest details beginning with this statement:<br><br>*I have read the journal's policy and the authors of this manuscript have the following competing interests: [insert competing interests here]*<br><br>* typeset	
**Ethics Statement**<br><br>Enter an ethics statement for this submission. This statement is required if the study involved:<br><br>• Human participants<br>• Human specimens or tissue<br>• Vertebrate animals or cephalopods<br>• Vertebrate embryos or tissues<br>• Field research<br><br>Write "N/A" if the submission does not require an ethics statement.<br><br>General guidance is provided below. Consult the submission guidelines for detailed instructions. **Make sure that all information entered here is included in the Methods section of the manuscript.**	Ethical approval is obtained from the Institutional Review Committee of NAIHS and is attached in a supplementary file. All the participants were informed about the study and its objective via online session before the study also incorporating the consent form in the questionnaire itself. Anyone who filled the form was understood to have given the consent

Powered by Editorial Manager® and ProduXion Manager® from Aries Systems Corporation

**Format for specific study types**<br><br>**Human Subject Research (involving human participants and/or tissue)**<br>• Give the name of the institutional review board or ethics committee that approved the study<br>• Include the approval number and/or a statement indicating approval of this research<br>• Indicate the form of consent obtained (written/oral) or the reason that consent was not obtained (e.g. the data were analyzed anonymously)<br><br>**Animal Research (involving vertebrate animals, embryos or tissues)**<br>• Provide the name of the Institutional Animal Care and Use Committee (IACUC) or other relevant ethics board that reviewed the study protocol, and indicate whether they approved this research or granted a formal waiver of ethical approval<br>• Include an approval number if one was obtained<br>• If the study involved *non-human primates*, add *additional details* about animal welfare and steps taken to ameliorate suffering<br>• If anesthesia, euthanasia, or any kind of animal sacrifice is part of the study, include briefly which substances and/or methods were applied<br><br>**Field Research**<br><br>Include the following details if this study involves the collection of plant, animal, or other materials from a natural setting:<br>• Field permit number<br>• Name of the institution or relevant body that granted permission	
**Data Availability**<br><br>Authors are required to make all data underlying the findings described fully available, without restriction, and from the time of publication. PLOS allows rare exceptions to address legal and ethical concerns. See the PLOS Data Policy and FAQ for detailed information.	Yes - all data are fully available without restriction

Powered by Editorial Manager® and ProduXion Manager® from Aries Systems Corporation

A Data Availability Statement describing where the data can be found is required at submission. Your answers to this question constitute the Data Availability Statement and **will be published in the article**, if accepted.<br><br>**Important:** Stating 'data available on request from the author' is not sufficient. If your data are only available upon request, select 'No' for the first question and explain your exceptional situation in the text box.<br><br>Do the authors confirm that all data underlying the findings described in their manuscript are fully available without restriction?	
**Describe where the data may be found in full sentences. If you are copying our sample text, replace any instances of XXX with the appropriate details.**<br><br>• If the data are **held or will be held in a public repository**, include URLs, accession numbers or DOIs. If this information will only be available after acceptance, indicate this by ticking the box below. For example: *All XXX files are available from the XXX database (accession number(s) XXX, XXX.*).<br>• If the data are all contained **within the manuscript and/or Supporting Information files**, enter the following: *All relevant data are within the manuscript and its Supporting Information files.*<br>• If neither of these applies but you are able to provide **details of access elsewhere**, with or without limitations, please do so. For example:<br><br>*Data cannot be shared publicly because of [XXX]. Data are available from the XXX Institutional Data Access / Ethics Committee (contact via XXX) for researchers who meet the criteria for access to confidential data.*<br><br>*The data underlying the results presented in the study are available from (include the name of the third party*	within the manuscript and/or Supporting Information files

and contact information or URL).<br>• This text is appropriate if the data are owned by a third party and authors do not have permission to share the data.<br><br>* typeset	
Additional data availability information:	

Manuscript

Click here to access/download;Manuscript;Hepatitis B vaccination status and KAP.docx

Hepatitis B vaccination status and knowledge, attitude, and practice regarding Hepatitis B among preclinical medical students of a medical college in Nepal

Dhan Bahadur Shrestha[1*], Manita Khadka[2], Manoj Khadka[2], Prarthana Subedi[2], SubashChandra Pokharel[2], Bikash Bikram Thapa[2]

[1]Mangalbare Hospital, Morang, Nepal

[2]Nepalese Army Institute of Health Sciences (NAIHS), Shree Birendra Hospital, Chhauni, Kathmandu, Nepal

Short title: Knowledge, attitude, and practice regarding Hepatitis B among medical students

[*]**Corresponding author**

E-mail: medhan75@gmail.com

Abstract

Background

Hepatitis B imposed a major public health problem with an increased risk of occupational exposure among health care workers. Medical students are also at increased risk due to a lack of mandatory vaccination programs and accidental needle stick injuries during clinical years. This demands the need for Hepatitis B vaccination of medical students before they enter clinical years. This study was conducted to determine the Hepatitis B vaccination status along with the knowledge, attitude, and practice regarding Hepatitis B among preclinical medical students of a medical college in Nepal.

Materials and Methods

This descriptive cross-sectional study was conducted among first and second-year medical students of a medical college in Kathmandu, Nepal from 6th July to 14th July 2020. The whole sampling technique was used. Data were collected using a pretested, self-administered questionnaire which was mailed to individuals and analyzed in the statistical package for social sciences version-22.

Results

A total of 181 students participated in the study out of 198, giving a response rate of 91.4%. Among the study participants, only 67 (37%) were fully vaccinated against Hepatitis B while 71 (39.2%) were never vaccinated. For the majority (74.6%) of the non-vaccinated participants, the main reason for not getting vaccination was a lack of vaccination programs. Regarding the knowledge,

attitude, and practice, half the study participants (n=92, 50.8%) had good knowledge, attitude and practice regarding hepatitis B. The median knowledge, attitude and practice score towards Hepatitis B were 61.00 (57.00-66.00), 20(18.00- 21.00) and 21(19.00- 23.00) respectively.

Conclusions

The majority of preclinical medical students were not fully vaccinated against Hepatitis B and only half of them, having acceptable knowledge, attitude and practice towards hepatitis B, which makes them vulnerable to hepatitis B during clinical years. This might represent the scenario of not only Nepal but South Asian countries and creates an alarm to taking the vaccination programs seriously.Since the majority of the non-vaccinated participants responded unavailability of vaccination program as the main cause, we strongly recommend the provision of the Hepatitis B vaccination program to the pre-clinical medical students so that they will be protected when they enter clinical training.

Keywords - Attitude; Hepatitis B; Knowledge; Medical students; Nepal; Practice; Vaccination

Introduction

Hepatitis B virus is the most contagious blood-borne pathogen that can cause both acute and chronic liver diseases [1,2]. Vaccination is the mainstay of prevention against Hepatitis B infection with 90%-100% protection conferred following the complete vaccination [2,3]. World Health Organization (WHO) recommends special consideration of healthcare workers & medical students for Hepatitis B virus screening and vaccination [2].

The risk of accidental exposure among medical students is high due to a lack of experience and direct contact with a patient's potentially infectious body fluids [4, 5, 6]. Hence, medical students with the start of clinical years are at high risk of Hepatitis B infection. However, no studies in the past assessed the Hepatitis B vaccination status among preclinical level medical students in Nepal.

To provide a basis for the implementation of Hepatitis B vaccination programs, our study aims to estimate the Hepatitis B vaccination status along with knowledge, attitude and practice regarding Hepatitis B infection among preclinical medical students of a medical college in Nepal.

Materials and methods

Study design and settings

This is a descriptive cross-sectional study done among the preclinical year (1st and 2nd year) students of the Nepalese Army Institute of Health Sciences (NAIHS), Nepal. The data for the study was collected from 6th July 2020 to 14th July 2020. The whole sampling technique was used. The students who were no longer attending classes and were not in contact were excluded from the study. This made the sample size to be 198 (first year-100, second year-98). The data was collected from the participants via Google forms circulated through the mail after explaining the objective of the study in the form itself. The participation was completely voluntary and the anonymity was insured. Those who submitted the response were considered to have given the consent. The participants didn't receive any incentives.

Study sample

The total population of the preclinical year of NAIHS was taken. The data collection time lasted from 6th July 2020 to 14th July 2020. All of the participants were reached in 4 phases by making 8 groups of 25 people each; through online sessions regarding study details. The expected non-response rate was kept at 5% of the total population of the study. During this survey period, all students were stranded at home due to the COVID-19 (Coronavirus disease 2019) pandemic imposed lockdown. Also Nepal government and global health governing agencies like WHO were advocating to limit gathering and movements due to fear of the spread of COVID. So we maile[illegible] questionnaire to participants via Google forms after an online session.

Study instrument

A self-administered online questionnaire containing 21 items was used for the study (supplementary file). It contained 3 items for demographics, 5 for the Knowledge section, 5 for the attitude and 5 for practice, 1 for vaccination status and 1 for total dose taken, and 1 for the reason of not getting vaccinated. The questionnaire was developed after an extensive literature search in the English language. The questionnaire was pre-tested among 5 % of the study sample, 10 students from the 3rd and 4th year of medical school, and modified accordingly. The questionnaire was then administered [illegible]r the necessary modification.

[illegible]xposure variables

The survey questionnaire included baseline exposure variables like age (below 19 years, 20 years and above), sex, vaccination status, and academic year (1st year, 2nd year).

Outc[illegible]e variables

A semi-structured pre-piloted knowledge, attitude, and practice questionnaire used to collect data, and its internal consistency was assessed by using Cronbach's α. The internal reliability of the present study found to be 0.698 indicating high internal consistency for our scale for this specific sample. Five-point Likert scale used to quantify the response on knowledge, attitude, and practice ranging from 1-5; 1 being the least acceptable response to the item asked while 5 being the most acceptable response to that item.

Statistical methods

The data collected through the google forms were extracted to Microsoft Excel-13 and was then imported and analyzed by using SPSS (Statistical Package for Social Sciences) version 22. Kolmogorov-Smirnov (K-S) test, the Shapiro-Wilk test was used to assess the normality of data distribution and classified data as normal if the significant value of the test is greater than 0.05. If the value is below 0.05, the data are classified as non-normal distribution. Our data were non-normal so non-parametric tests were used using the median as a measure of distribution. A Chi-square test was used to check the association between variables. Spearman's rho was used to check the correlation between knowledge and attitude, knowledge and practice and attitude, and practice.

Ethical consideration

The study was approved by the Institutional Review Committee of NAIHS. All the participants were informed about the study and its objective by incorporating the consent form in the questionnaire itself. Anyone who filled the form was understood to have given the consent.

Results

General characteristics

Table 1 shows the general characteristics of the respondents who participated in the study. Out of 198 preclinical medical students, 181 (91.4% response rate) students participated in the study, 85 (47%) from the first year, and 96 (53%) from the second year. The mean age of the study participants was 19.93 (±1.436) years, ranging from 17-28 years. The majority of the respondents were male (n=123, 68%) ~~as~~ compared to females (n=58, 32%). Regarding Hepatitis B vaccination status, 71 (39.2%) study participants weren't vaccinated while 110 (60.8%) students were vaccinated. Among the 110 vaccinated students, 67 (60.9%) received at least 3 doses of Hepatitis B while 43 (39.1%) received less than 3 doses. Among 71 non-vaccinated students, the major reason for not being vaccinated was the lack of a vaccination program being offered (n=53, 74.6%).

Table 1. Baseline characteristics and Hepatitis B vaccination status of the respondents (N = 181).

Variables		n	%
Age (in Years)	19 and below	67	37.0
	20 and above	114	63.0
	Mean±SD	19.93±1.436	
Gender	Male	123	68.0
	Female	58	32.0
Academic year	1st Year MBBS	85	47.0
	2nd Year MBBS	96	53.0
Vaccinated against hepatitis B	No	71	39.2
	Yes	110	60.8

Doses of Hepatitis B vaccine received	Not vaccinated	71	39.2
	One	14	7.7
	Two	29	16.0
	Three	55	30.4
	More than three	12	6.6
Reason for Not vaccinate[illegible] against Hepatitis B	No vaccination program offered	53	29.3
	Low risk of Hepatitis B	6	3.3
	Not sure about vaccination status	4	2.2
	Lack of Knowledge	4	2.2
	High vaccination fees	2	1.1
	Efficacy doubted	2	1.1

Assessment of Knowledge related to Hepatitis B

The majority of the study participants agreed that Hepatitis B is caused by a virus (87.3% strongly agreed) and Hepatitis B can cause liver cancer (43.6% strongly agreed, 37% agreed). In terms of knowledge on the mode of transmission, around three four[illegible]f the respondents strongly agreed on contaminated blood and body fluids (78.5%), unsterilized syringes/needles (75.7%), unprotected sex (71.8%) and infected mother to fetal transmission (71.8%). Similarly, most of the respondents disagreed on casual contact (49.2% strongly disagreed, 26.5% disagreed) and cough/sneeze (40.9[illegible]rongly disagreed, 22.7% disagreed) as a mode of transmission. However, regarding contaminated food/water as a mode of transmission for Hepatitis B, only 33.7% strongly disagreed while 22.1% were neutral and 11.6% strongly agreed. About two-[illegible]rd agreed that

healthcare workers are at increased risk of getting Hepatitis B (29.8% strongly agreed, 38.1% agreed). When asked about prevention, the majority responded with vaccination (80.1% strongly agreed), avoiding sharp needles/syringes (39.8% strongly agreed, 42% agreed), and using gloves when handling body fluids (47% strongly agreed, 42% agreed). However, regarding antivirals as a preventive measure, only 3.3% strongly disagreed while 24.9 % were neutral and 26.5% strongly agreed. Additionally, 20.4% strongly disagreed on avoiding contaminated food/water as a preventive measure for Hepatitis B while25.4% were neutral and 11.6% strongly agreed **(Table 2)**. The median score for knowledge was 61 **(Table 3)**.

Table 2. Knowledge, Attitude and Practice findings of the respondents.

Questions for response	Strongly Disagree n(%)	Disagree n (%)	Neutral n (%)	Agree n (%)	Strongly agree n(%)
**Assessment of Knowledge related to Hepatitis B**					
1. Hepatitis B is caused by a virus.	10 (5.5)	4 (2.2)	0 (0)	9 (5.0)	158 (87.3)
2. Hepatitis B can be transmitted by :<br>a. Infected mother to fetus	2 (1.1)	3 (1.7)	9 (5.0)	37 (20.4)	130 (71.8)
2. b. Contaminated blood and body fluids	2 (1.1)	1 (0.6)	2 (1.1)	34 (18.8)	142 (78.5)
2. c. Unprotected sex	7 (3.9)	5 (2.8)	10 (5.5)	29 (16.0)	130 (71.8)
2. d. Casual contact (shaking hands)	89 (49.2)	48 (26.5)	18 (9.9)	16 (8.8)	10 (5.5)
2. e. Unsterilized syringes/needles	3 (1.7)	2 (1.1)	4 (2.2)	35 (19.3)	137 (75.7)
2. f. Coughing/sneezing	74 (40.9)	41 (22.7)	33 (18.2)	19 (10.5)	14 (7.7)
2. g. Contaminated food/water	61 (33.7)	30 (16.6)	40 (22.1)	29 (16.0)	21 (11.6)
3. Hepatitis B can cause liver cancer.	4 (2.2)	8 (4.4)	23 (12.7)	67 (37.0)	79 (43.6)
4. Healthcare workers are at increased risk of getting hepatitis B than general population:	5 (2.8)	16 (8.8)	37 (20.4)	69 (38.1)	54 (29.8)
5. Hepatitis B can be prevented by:<br>a. Vaccination	2 (1.1)	2 (1.1)	3 (1.7)	29 (16.0)	145 (80.1)
5. b. Antivirals	6 (3.3)	7 (3.9)	45 (24.9)	75 (41.4)	48 (26.5)

5. c. Avoiding sharp needle/syringe injury	5 (2.8)	10 (5.5)	18 (9.9)	76 (42.0)	72 (39.8)
5. d. Avoiding contaminated food/water	37 (20.4)	34 (18.8)	46 (25.4)	43 (23.8)	21 (11.6)
5. e. Using gloves when handling body fluids	1 (0.6)	5 (2.8)	14 (7.7)	76 (42.0)	85 (47.0)
**Assessment of Attitude towards Hepatitis B**					
6. I feel uncomfortable sitting with a hepatitis B infected person.	34 (18.8)	45 (24.9)	55 (30.4)	35 (19.3)	12 (6.6)
7. I don't mind shaking hands/hugging with a hepatitis B infected person.	12 (6.6)	26 (14.4)	41 (22.7)	65 (35.9)	37 (20.4)
8. I believe the hepatitis B vaccine is safe and effective.	5 (2.8)	2 (1.1)	17 (9.4)	92 (50.8)	65 (35.9)
9. I believe healthcare workers should receive hepatitis B vaccination.	0 (0)	0 (0)	13 (7.2)	49 (27.1)	119 (65.7)
10. I don't need hepatitis B vaccination because I am not at risk	101 (55.8)	54 (29.8)	19 (10.5)	6 (3.3)	1 (0.6)
**Assessment of Practice towards Hepatitis B**					
11. I ask/use a new blade for shaving/hair cutting.	4 (2.2)	4 (2.2)	11 (6.1)	33 (18.2)	129 (71.3)
12. I ask for a new syringe before injection.	0 (0)	1 (0.6)	6 (3.3)	22 (12.2)	152 (84.0)
13. I ask for sterilized equipment for ear/nose piercing.	3 (1.7)	2 (1.1)	18 (9.9)	48 (26.5)	110 (60.8)
14. I always report for needle prick / sharp injuries.	0 (0)	18 (9.9)	46 (25.4)	59 (32.6)	58 (32.0)
15. I attend hepatitis B related awareness programs.	15 (8.3)	21 (11.6)	65 (35.9)	50 (27.6)	30 (16.6)

Table 3. Summation of knowledge, attitude and practice score distribution

	Knowledge sum (n=181)	Attitude sum(n=181)	Practice Sum(n=181)	Total Score (n=181)
Mean	60.86	19.91	20.97	101.73

Median	61.00	20.00	21.00	102.00
IQR	(57.00-66.00)	(18.00- 21.00)	(19.00- 23.00)	(96.00- 108.00)

Assessment of Attitude towards Hepatitis B

The majority were neutral (n=55, 30.4%) to sitting with a Hepatitis B positive person, however, 6.6% (n=12) strongly agreed on feeling uncomfortable while sitting with a Hepatitis B positive person and 18.8% (n=34) strongly disagreed. Many don't mind shaking hands with a Hepatitis B positive person (n=65, 35.9 %), however, 6.6 % (n=12) strongly disagreed on doing so. Around half of the respondents, 50.8% (n=92) agreed that hepatitis B vaccination is safe and effective whereas 2.8% (n=5) strongly disagreed. Most of the respondents, 65.7% (n= 119) strongly agreed that healthcare workers should receive hepatitis B vaccination and 55.8% (n=101) strongly agreed that they need Hepatitis B vaccination because they are at risk **(Table 2)**. The median attitude score was 20 **(Table 3)**

Assessment of Practice towards Hepatitis B

Among the respondents, the majority (n=129, 71.3%) ask for a new blade while cutting or shaving hair. 84% (n=152) of them ask for a new syringe before injection. 32% (n=58) strongly agreed on reporting their needle prick injury whereas 9.9% (n=18) disagreed. A small number of people (n=15, 8.3%) strongly disagreed on attending any hepatitis B awareness program whereas 27.6% (n=50) agreed **(Table 2)**. The median score for practice was 21 **(Table 3)**.

Categorization of Knowledge Attitude and Practice (KAP) score and its association with baseline characteristics

Table 4 demonstrates the association of KAP score with baseline characteristics like age, gender, academic year, vaccination status, doses of vaccination received, and reasons for not being vaccinated. There is no significant association observed. The median KAP score was 102 **(Table 3)**. **Fig 1** shows that the KAP was good (above the median score) among 92 (50.8%) respondents.

Table 4. Association of KAP score with baseline characteristics.

Variables		KAP score Category		p-value
		In-adequate (<102)	Good (≥102)	
Gender:	Male	62	61	.628
	Female	27	31	
Age	19 and below	36	31	.347
	20 and above	53	61	
Academic year	1st Year MBBS	44	41	.511
	2nd Year MBBS	45	51	
Vaccinated against hepatitis B	No	37	34	.525
	Yes	52	58	
Doses of Hepatitis B vaccine	No	37	34	.455
	One	8	6	
	Two	10	19	
	Three	29	26	
	More than three	5	7	
Reason for Not vaccinated against Hepatitis B	No vaccination program offered	27	26	.564
	Low risk of Hepatitis B	3	3	
	Not sure about vaccination status	1	3	
	High vaccination fees	2	0	
	Efficacy doubted	1	1	

	Lack of Knowledge	3	1	

Figure 1. KAP score category among the respondents.

Correlation of knowledge, attitude, and practice on Hepatitis B

We found a weak positive correlation between knowledge with attitude (r=0.343) and attitude with practice (r=0.170). However, no significant correlation was found between knowledge with practice (r=0.009, p-value 0.909) **(Table 5)**.

Table 5. Correlation of knowledge, attitude, and practice.

Variables	Spearman's correlation coefficient	p-value
Knowledge-Attitude	0.343*	p< 0.01
Knowledge-Practice	0.009	P=0.909
Attitude-Practice	0.170**	P=0.022

*Correlation is significant at p< 0.01 level (2-tailed).

**Correlation is significant at the 0.05 level (2-tailed).

Discussion

The risk of exposure to Hepatitis B infection among medical students is the same, if not greater [illegible] other healthcare workers since they are banked on to be involved in patient care at the beginning

of clinical training. This highlights the importance of getting vaccinated and acquiring adequate knowledge about the infection before getting into clinical training. However, Nepal lacks adequate studies that assess the vaccination status of the students and their knowledge, attitude, and practice regarding Hepatitis B infection. This study looks into the vaccination status of preclinical students and describes their KAP towards the infection. These students will soon get exposed to clinical settings after the COVID-19 pandemic will come under control.

The Hepatitis B vaccination status among preclinical medical students in our study was 60.8% which is quite lower than the only other study done among medical students in the country where 86.5% of the students were vaccinated [7]. But our finding is similar to a study from Pakistan, where 60% were vaccinated [8]. And it is higher than the findings of a study from Nigeria and another study from Pakistan where 47.7% and 42.2% were reported to have been vaccinated respectively [9,10]. Among vaccinated students, 60.9% were fully vaccinated (3 and more doses). This is similar to the study from Saudi Arabia where 38% were found to have received all three doses and much higher than 2% completing all three doses according to a finding from Ethiopia [11,12]. However, the finding from another study done Nepal showed a higher percentage (83.7%) of students completing full doses [7]. This warrants a need to look into the vaccination status of students before going into clinical years to ensure high vaccination rates during their clinical training.

Among the non-vaccinated participants (39.2%) of our study, the main reasons for non-vaccination were found to be: no vaccination program offered (74.6%) followed by a low risk of Hepatitis B (8.5%) and lack of knowledge (5.6%). The result is similar to other studies from Nepal and Nigeria that showed a lack of vaccination programs (43.2%) and lack of opportunity (57.4%) respectively as the major reasons for non-vaccination [7,9]. These findings sufficiently shed light on the urgent

necessity to implement vaccination programs for medical students. However, the high cost of vaccines was stated as the major reason for non-vaccination among health science students from Uganda [13]. A study from Pakistan showed non-compliance as a major reason for the low -vaccination rate [8].

Out of the students surveyed, 92.3% were aware that Hepatitis B is caused by a virus. A study from Nepal done among preclinical medical and dental students revealed that 93.6% of the participants were aware of the cause of Hepatitis B infection [14]. Regarding the knowledge about the mode of transmission, the majority knew about infected mother to fetus transmission (92.2%), transmission through contaminated blood and body fluids (97.3%), unprotected sex (87.8%), and unsterilized syringes/needles (95%). The findings are parallel to the findings of a study from Northeast Ethiopia and higher than the study from Haramaya University, Ethiopia [12,15]. However, very few participants strongly disagreed with the transmission of infection via casual contact, coughing/sneezing, and contaminated food/water. This gap in knowledge could increase the probability of patients with hepatitis B infection from being ostracized from society

80.6% of respondents agreed Hepatitis B virus can cause liver cancer in the present study. This is comparable with Ethiopian and Saudi Arabian study among medical students showing 81.3 % and 75.5% agreeing hepatitis B causes liver cancer respectively. [11, 12]. 96.1% of our study participants agreed that the Hepatitis B infection can be prevented by vaccination. A study by Alhowaish MA et al and Abdela A et al showed 86.5% and 84.6% respectively responded vaccine prevents hepatitis B [11,12].

Regarding attitude related to Hepatitis B infection and vaccination; 43.7% didn't feel uncomfortable sitting with a Hepatitis B positive person. 56.3% didn't mind shaking hands or hugging with a Hepatitis B infected person which is higher than the finding from Saudi Arabia

indicating the more acceptable attitude of students responding to the present study [16]. In our study, 86.7% of the participants thought that the hepatitis B vaccine was safe and effective which is similar to a finding from Saudi Arabia and Ethiopia [11,12]. This finding is higher as compared to another study from Saudi Arabia where only 63% considered the vaccine safe [16]. 92.8% of our participants believed that healthcare workers should be vaccinated which is notably higher as collated with a study from Saudi Arabia [16]. The same study from Saudi Arabia also stated that 62% of its participants believed that they were at higher risk of contracting Hepatitis B infection; which is lower than our study's finding which 85.6% [16]. As of our study, 3.9% thought that they don't need to be vaccinated and that they are not at risk which is comparable with an Indian study's finding with 3.7% responding they don't need it [17]. Though this is a small proportion as compared to other findings it needs to be addressed because medical students, being a part of the healthcare delivery system, should be well aware that they need to be vaccinated as they are always at risk of contracting and spreading the Hepatitis B infection.

In our study, 89.5%, 96.2%, and 87.3% of the participants asked for a new blade for shaving/hair cutting, a new syringe for injection, and sterile equipment for ear/nose piercing respectively which reflects the good safety practices among the participants. A study by Rathi A et al also showed similar findings in the case of participants asking for a new blade for shaving/hair cutting and sterile equipment for nose/ear piercing [17]. The finding for asking new syringes for injection in our study is more than that in Ethiopia and India [15,17]. 64.6% of our study participants said that they always report for needle prick/sharp injuries which is similar to Alhowaish MA et al showing 68% while a study by Abdela A et al reported only 53.7% will report needle stick injury[11, 12]. 44.2% of our study participants agreed that they attend Hepatitis B related awareness programs which are significantly greater than the finding from Ethiopia i.e. 23.9% [15].

This study found that females had better overall KAP scores compared to males which are per the previous study from Pakistan [18]. However, it contradicts the study from Malaysia which had found no association between gender and knowledge [19]. Similarly, second-year students had better KAP scores than first-year students which are in line with the findings of a study from Pakistan where 1st professional year had the least knowledge [18]. This can be associated with the fact that second-year students have got more instructions and information and are more aware of the disease. Those who had been vaccinated were found to have better KAP scores than those who weren't. A weak positive correlation was found between knowledge with attitude and attitude with practice somewhat similar to the correlations found in a study from Malaysia [19].

The major limitation of this study is that we didn't measure the anti- Hepatitis B surface antibody (HBsAb) titer of the participants; hence the vaccination status could not be verified. Likewise, recall bias and information bias might have also affected the result of our study. We had mentioned the date of the introduction of the Hepatitis B vaccine in the immunization program of Nepal in the questionnaire and also collected the responses anonymously to mitigate these biases. This study was done by collecting responses using an online questionnaire in a country where internet penetration is 57% [20]. This may have affected the response rate of our study. Finally, the results may not be generalizable to all the medical colleges of Nepal since the data was collected only from NAIHS but it will surely provide a reference for further research in this field. The health care system needs to be strengthened in poor resource countries like Nepal where a limited number of health care providers are available. Due to the limited availability of resources, health protection, and promotion of providers has to be kept under consideration. Due to the COVID-19 pandemic, the health care delivery system is highly impacted including clinical learning of medical students. Before they get exposed to the clinical rotation, we recommend the governing body to make

available and implement mandatory Hepatitis B vaccination to safeguard the health of medical students.

Conclusion

We found that there were more than one-fourth of the respondents not vaccinated for Hepatitis B despite it being a vaccine-preventable disease. The main reason for not getting vaccinated was the unavailability of vaccination programs. Vaccination programs need to be provided for all healthcare workers and medical students before they go into their clinical years so that they can prev[illegible] themselves from the hazards of non-vaccination and needle stick injuries. A change must be instilled from the regulatory bodies of medical schools as well as the government and a free vaccination program can be administered to all students coming into the hospital before they enter clinical years.

Declarations

Ethical approval and consent to participate:

Ethical approval is obtained from the Institutional Review Committee of NAIHS and is attached in a supplementary file. All the participants were informed about the study and its objective via online session before the s[illegible]y also incorporating the consent form in the questionnaire itself. Anyone who filled the form was understood to have given t[illegible]onsent

Consent for Publication: not applicable

Availability of data and materials: The datasets analyzed during the current study are available from the corresponding author on reasonable request.

Competing interests: The authors declare that they have no competing interests.

Funding: This article did not receive any specific grant from funding agencies in the public, commercial, or any other sectors.

Authors' contributions

DBS, MK, MK, PS, SP, and BBT contributed to concept and design, literature search, data collection, interpretation of data, and review. DBS analyzed data.

All authors were involved in drafting and revising the manuscript and approved the final version.

“All authors listed have read and approved the manuscript” for further processing and publication.

Acknowledgements

Not applicable

Authors' information

DBS Medical officer, Department of Emergency Medicine, Mangalbare Hospital, Morang, Nepal

Email: medhan75@gmail.com

ORCid:0000-0002-8121-083X

MK medical student, Nepalese Army Institute of Health Sciences (NAIHS), Kathmandu, Nepal

Email: khadkamanita22@gmail.com

ORCiD:0000-0001-9263-2598

MK medical student, Nepalese Army Institute of Health Sciences (NAIHS), Kathmandu, Nepal

Email:khadkamanoj432@gmail.com

ORCiD: 0000-0002-0449-9254

PS medical student, Nepalese Army Institute of Health Sciences (NAIHS), Kathmandu, Nepal

E-mail: prarthana.subedi.ps@gmail.com

ORCiD: 0000-0002-3188-0878
SP medical student, Nepalese Army Institute of Health Sciences (NAIHS), Kathmandu, Nepal
E-mail: sc.pokharel3@gmail.com
ORCiD: 0000-0003-1523-6158
BBT assistant professor, Department of Surgery, Nepalese Army Institute of Health Sciences (NAIHS), Shree Birendra Hospital, Chhauni, Kathmandu, Nepal
Email: bikashbikram.thapa@naihs.edu.np / bbthapa@gmail.com
ORCiD: 0000 0001-7513-8854

References

1. CDC - NIOSH Publications and Products - Preventing Needlestick Injuries in Health Care Settings (2000-108) [Internet]. [cited 2020 May 15]. Available from: https://www.cdc.gov/niosh/docs/2000-108/default.html

2. Hepatitis B [Internet]. [cited 2020 May 15]. Available from: https://www.who.int/en/news-room/fact-sheets/detail/hepatitis-b

3. Safary A, Andre FE. Clinical experience with a yeast-derived hepatitis B vaccine.J Chemother. 1989;1(4 Suppl):1096–8. DOI: https://doi.org/10.1016/0264-410X(90)90222-8

4. Kavitha N, Kumar K, Bhardwaj A, Muthalagan D, Yhmin C, Lun L, et al. Prevalence Of Needle Stick Injuries And Their Underreporting Among Healthcare Workers In The Department Of Obstetrics And Gynaecology. Int Arch Med. 2015 Jan 1; DOI: http://dx.doi.org/10.3823/1780

5. Ibrahim N, Idris A. Hepatitis B Awareness among Medical Students and Their Vaccination Status at Syrian Private University. Hepat Res Treat. 2014;2014:1–7. DOI: https://doi.org/10.1155/2014/131920

6. Deisenhammer S, Radon K, Nowak D, Reichert J. Needlestick injuries during medical training. J Hosp Infect. 2006;63(3):263–7. DOI: https://doi.org/10.1016/j.jhin.2006.01.019

7. Bhattarai S, Smrit KC, Pradhan PMS, Lama S, Rijal S. Hepatitis B vaccination status and Needle-stick and Sharps-related Injuries among medical school students in Nepal: A cross-sectional study. BMC Res Notes. 2014;7(1):1–7. DOI: https://doi.org/10.1186/1756-0500-7-774

8. Butt M, Khan IM, Ashfaq MW, Jamal M. Hepatitis B Vaccination Status among Students of a Medical College in Islamabad. J Islam Med Dent Coll [Internet]. 2015;20154(4):157–61. Available from: https://pdfs.semanticscholar.org/529f/dbf86ff258021ee26275af8b290e45ea7c34.pdf

9. Okeke EN, Ladep NG, Agaba EI, Malu AO. Hepatitis B vaccination status and needle stick injuries among medical students in a Nigerian university. Niger J Med. 2008;17(3):330–2. DOI: https://doi.org/10.4314/njm.v17i3.37404

10. Nasir K, Khan KA, Kadri WM, Salim S, Tufail K, Sheikh HZ, et al. Hepatitis B vaccination among health care workers and students of a medical college. J Pak Med Assoc. 2000;50(7):239–43. Available from: https://pubmed.ncbi.nlm.nih.gov/10992702/ PMID: 10992702

11. Alhowaish MA, Ali Alhowaish J, HamoudAlanazi Y, ManaAlshammari M, SaeidAlshammari M, GhadeerAlshamari N, et al. Knowledge, attitudes and practices

toward prevention of hepatitis B virus infection among medical students at Northern Border University, Arar, Kingdom of Saudi Arabia. Electron Physician [Internet]. 2017 Sep 25 [cited 2020 Jul 17];9(9):5388–94. DOI: https://doi.org/10.19082/5388

12. Abdela A, Woldu B, Haile K, Mathewos B, Deressa T. Assessment of knowledge, attitudes and practices toward prevention of hepatitis B virus infection among students of medicine and health sciences in Northwest Ethiopia. BMC Res Notes [Internet]. 2016 Aug 19 [cited 2020 Jul 17];9(1):410. DOI: https://dx.doi.org/10.1186%2Fs13104-016-2216-y

13. Wibabara Y, Banura C, Kalyango J, Karamagi C, Kityamuwesi A, Amia WC, et al. Hepatitis B vaccination status and associated factors among undergraduate students of Makerere University College of Health Sciences. PLoS One. 2019;14(4):1–9. DOI: https://doi.org/10.1371/journal.pone.0214732

14. Shah D, Jha R, Ansari S, Sah P, Dhungana G, Basnet S. Knowledge and awareness regarding hepatitis B among preclinical medical and dental students of Chitwan Medical College Nepal: a questionnaire-based study. Int J Med Sci Public Heal. 2016;5(11):2316. DOI: https://dx.doi.org/10.5455/ijmsph.2016.15042016474

15. Mesfin YM, Kibret KT. Assessment of knowledge and practice towards hepatitis B among medical and health science students in Haramaya University, Ethiopia. PLoS One [Internet]. 2013 Nov 21 [cited 2020 Jul 17];8(11). DOI: https://dx.doi.org/10.1371%2Fjournal.pone.0079642

16. Al-Hazmi A. Knowledge, attitudes, and practice of medical students regarding occupational risks of hepatitis B virus in College of Medicine, Aljouf University. Ann Med Health Sci Res [Internet]. 2015 [cited 2020 Jul 17];5(1):13. Available form:

https://www.ajol.info/index.php/amhsr/article/view/112493

17. Rathi A, Kumar V, Majhi J, Jain S, Lal P, Singh S. Assessment of knowledge, attitude, and practices toward prevention of hepatitis B infection among medical students in a high-risk setting of a newly established medical institution. J Lab Physicians [Internet]. 2018 Oct [cited 2020 Jul 17];10(04):374–9. DOI: https://dx.doi.org/10.4103%2FJLP.JLP_93_18

18. Khan N, Ahmed SM, Khalid MM, Siddiqui SH, Merchant AA. Effect of gender and age on the knowledge, attitude and practice regarding Hepatitis B and C and vaccination status of Hepatitis B among medical students of Karachi, Pakistan. Available from: https://jpma.org.pk/article-details/2098?article_id=2098

19. Ahmad A, Munn Sann L, Abdul Rahman H. Factors associated with knowledge, attitude and practice related to hepatitis B and C among international students of Universiti Putra Malaysia. BMC Public Health [Internet]. 2016 Jul 21 [cited 2020 Jul 17];16(1):611. DOI: https://doi.org/10.1186/s12889-016-3188-5

20. Govenment of Nepal Mocait. 2018 digital Nepal's network: unlocking Nepal's growth potential.: Government of Nepal; 2018. [cited 2020 Jul 17]. Available from: https://mocit.gov.np/application/resources/admin/uploads/source/EConsultation/Final%20 Book.pdf

Figure legends

Figure 1:KAP score category among the respondents

List of supplementary files

Supplementary file 1: Questionnaire

Figure

Click here to access/download;Figure;Figure 1. Hepatitis B KAP status.tiff

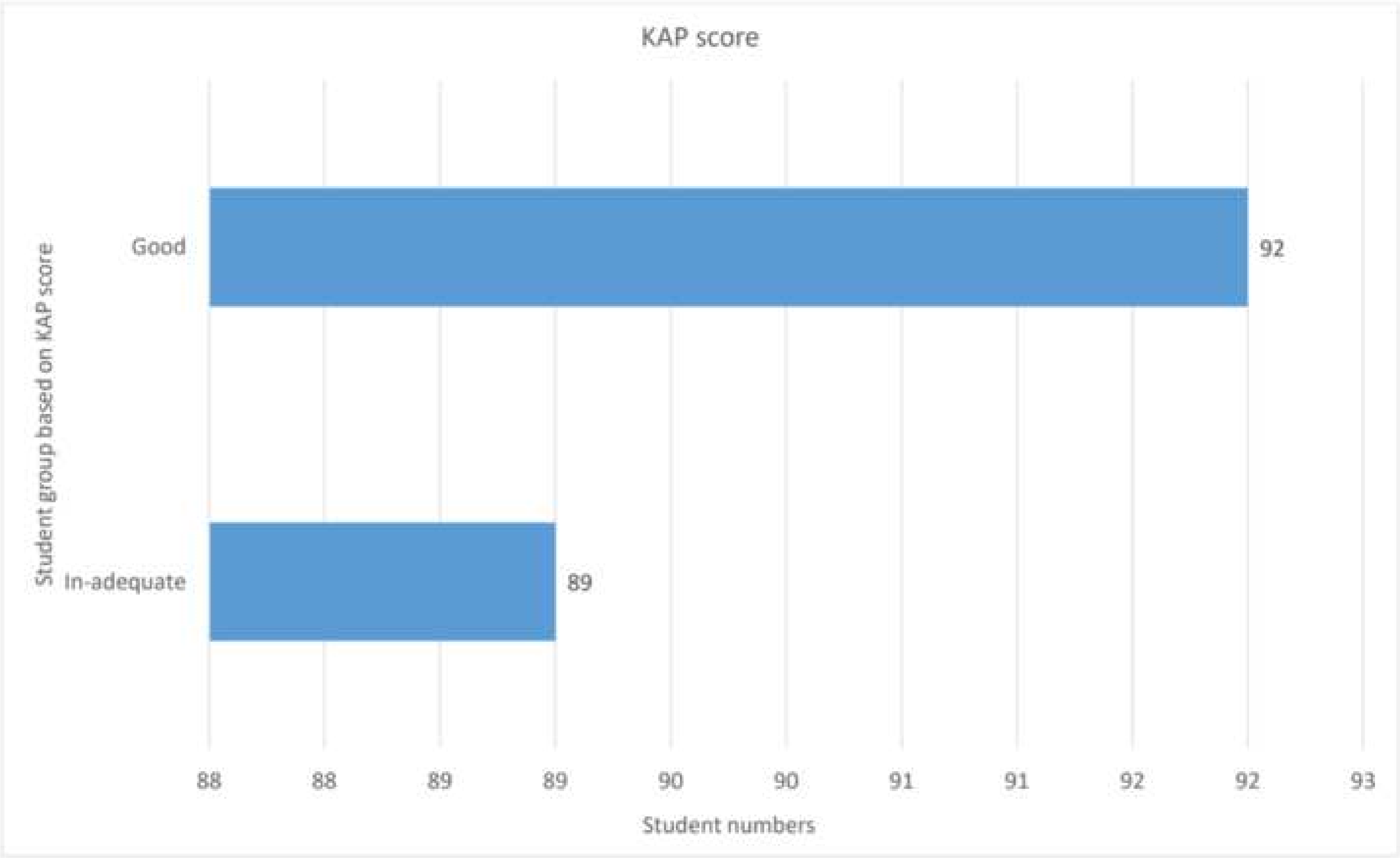

Click here to access/download
Supporting Information
Supplementary file HBV Questionnaire.docx
