# Supplementary material for: Hepatitis B vaccination status and knowledge, attitude, and practice regarding Hepatitis B among preclinical medical students of a medical college in Nepal
Source: PLoS One. 2020 Nov 23;15(11):e0242658. doi: 10.1371/journal.pone.0242658 (PMC7682811; doi:10.1371/journal.pone.0242658)
Supplement: Attachment — Submitted filename: Responses to Reviewer.docx [file pone.0242658.s004.docx]

Responses to Reviewer's comments and Questions

1) Please ensure that your manuscript meets PLOS ONE's style requirements, including those for file naming. The PLOS ONE style templates can be found at

<https://journals.plos.org/plosone/s/file?id=wjVg/PLOSOne_formatting_sample_main_body.pdf> and

<https://journals.plos.org/plosone/s/file?id=ba62/PLOSOne_formatting_sample_title_authors_affiliations.pdf>

Reply: PLOS ONE style followed

2) Your ethics statement should only appear in the Methods section of your manuscript. If your ethics statement is written in any section besides the Methods, please delete it from any other section.

Reply: Ethics statement removed from other area apart from Methods section

Reviewers' comments:

Comments to the Author

1. Is the manuscript technically sound, and do the data support the conclusions?

The manuscript must describe a technically sound piece of scientific research with data that supports the conclusions. Experiments must have been conducted rigorously, with appropriate controls, replication, and sample sizes. The conclusions must be drawn appropriately based on the data presented.

Reviewer #1: Yes

Reviewer #2: Yes

Reply: NA

2. Has the statistical analysis been performed appropriately and rigorously?

Reviewer #1: Yes

Reviewer #2: Yes

Reply: NA

3. Have the authors made all data underlying the findings in their manuscript fully available?

The PLOS Data policy requires authors to make all data underlying the findings described in their manuscript fully available without restriction, with rare exception (please refer to the Data Availability Statement in the manuscript PDF file). The data should be provided as part of the manuscript or its supporting information, or deposited to a public repository. For example, in addition to summary statistics, the data points behind means, medians and variance measures should be available. If there are restrictions on publicly sharing data—e.g. participant privacy or use of data from a third party—those must be specified.

Reviewer #1: Yes

Reviewer #2: No

Reply: All data collected were analyzed and made available in the manuscript. No part of data left un-analyzed

4. Is the manuscript presented in an intelligible fashion and written in Standard English?

PLOS ONE does not copyedit accepted manuscripts, so the language in submitted articles must be clear, correct, and unambiguous. Any typographical or grammatical errors should be corrected at revision, so please note any specific errors here.

Reviewer #1: Yes

Reviewer #2: No

Reply: Any language, typographical, presentation or grammatical errors commented and raised by reviewers revisited and rectified. Also all authors review papers to pick any such errors to avoid it.

5. Review Comments to the Author

Please use the space provided to explain your answers to the questions above. You may also include additional comments for the author, including concerns about dual publication, research ethics, or publication ethics. (Please upload your review as an attachment if it exceeds 20,000 characters)

Reviewer #1: This paper reports on a study examining Hepatitis B vaccination status and knowledge, attitudes and practices of Hepatitis B vaccination amongst medical students in Nepal. The manuscript would be strengthened by attention to the following:

1) In the introduction, provide more of an explanation about why it would be important to study medical students’ and their status, knowledge, etc. in Nepal. Does Nepal have a particularly high Hep B infection rate? Would students need to be informed to help provide education to their patients and communities? How would learning about this in the Nepalese context help others who do not live/work/educate students in Nepal. When was the Hep B vaccine introduced in Nepal?

Reply: These issues rectified by adding and modifying content in introduction section to give lucid idea of Hepatitis B in context of Nepal and its utility in other part of world.

2) I think in the methods the authors intend to state the survey was distributed through e-mail, since it was a Google form that was used.

Reply: All participants were informed and detailed about the aims of study via online group discussion dividing all two batches into smaller groups, after that all individuals were requested to fill form made available as Google form and link shared to individuals through email.

3) On page 5, there is reference to online sessions regarding study details – can you provide more information about what those were? Were they optional for students/study participants? What information was presented during the session, i.e., purpose of the study and an opportunity to ask questions?

Reply: Those session were conducted to provide detail information regarding study objectives and implications to the participants and to answer any questions related to study.

4) While the survey is in a supplemental file (which I could not reach), it would be helpful to provide some more detail about the nature of the questions asked and associated response formats. For the knowledge section, what were the topics, for the attitude section, what were the attitudes/topics, and similarly for the practice questions? What were the options for not getting vaccinated? What were the demographic questions?

Reply: Questions were made available as supplemental file and those questions were analyzed and kept in tables of result sections respectively.

5) With respect to the discussion of the findings, would not the medical school curriculum present information about how Hep B is transmitted to students? The respondents in general appear knowledgeable about the cause and mode of transmission, however, given their education as future physicians, I would hope they would all get those questions “correct.” When infectious disease is taught in the curriculum and is Hep B a particular topic?

Reply: Based on curriculum of Microbiology subject in Tribhuvan university (where NAIHS is affiliated), during first and second year of preclinical days infectious disease is taught to medical students.

6) In the discussion and conclusion, address why a reader outside of Nepal would be interested/need to know this information. This relates back to the first suggestion. The authors do include information about studies from other countries in the discussion which is a strength of the discussion. The authors also advocate that vaccination programs should be available for health care workers. While such advocacy is clearly at the national level, would it be helpful if global organizations, such as the World Health Organization, advocated this and/or perhaps provided resources for these programs? I don’t know – I am just speculating and trying to help the authors have their work make more of an impact than at the Nepalese national level.

Reply: Relevant discussion in introduction, discussion and conclusion section added/modified to address above issues. Also at present tourism and global migration is increased so it is advisable to get vaccinated for Hep B among high risk groups.

Reviewer #2: I noted a great deal of simply awkward word choices, and corrected most of them in a copy of the pdf that I added "comments" to. This is not how I usually do it. (I usually copy the sentence and propose the changes in a separate word file, which I then paste the contents of in this box.)

But since I was using that format, I also made 3 or 4 remarks that require some additional writing. I will sommarize them here.

1. Acknowledge that your respondents probably wanted to look good in your eyes, and their teachers' eyes, and their own eyes. "social desirability". So instead of saying "x% of them report needle sticks", say "x% of them SAY THAT they report needle sticks." for example.

Reply: Implemented and rectified as appropriate.

I don't know what the right answers to the knowledge test are, what appropriate attitudes are for a physician, and what the recommended behaviors are. You do not identify in the text which items are true and which false, and you do not discuss how you treat the numerical scores on those items. I hope you did not add them all up, true and false together!

Reply: These issues discussed in more detail and in lucid ways in method section.

You present some summary data - sums of the ratings on items about attitudes, about knowledge. But you do not say what the maximum possible score would be. Are these numbers comparable? The reader cannot judge if students are good at knowledge, but poor at translating the knowledge into habitual practice (for example), if you don't say the ranges of the scales. Also, you report an overall scale that combines knowledge, attitude, and behavior into one numerical scale. How should we interpret that? Are they equally weighted? on what basis?

Reply: We authors found that score scale were missing so added in method section while revising the draft.
