# Supplementary material for: Hepatitis B vaccination status and knowledge, attitude, and practice regarding Hepatitis B among preclinical medical students of a medical college in Nepal
Source: PLoS One. 2020 Nov 23;15(11):e0242658. doi: 10.1371/journal.pone.0242658 (PMC7682811; doi:10.1371/journal.pone.0242658)
Supplement: Attachment — Submitted filename: Response to reviewer.docx [file pone.0242658.s005.docx]

Response to reviewer

**Comments to the Author**

1. If the authors have adequately addressed your comments raised in a previous round of review and you feel that this manuscript is now acceptable for publication, you may indicate that here to bypass the “Comments to the Author” section, enter your conflict of interest statement in the “Confidential to Editor” section, and submit your "Accept" recommendation.

Reviewer #2: (No Response)

Authors Reply: Not applicable

2. Is the manuscript technically sound, and do the data support the conclusions?

The manuscript must describe a technically sound piece of scientific research with data that supports the conclusions. Experiments must have been conducted rigorously, with appropriate controls, replication, and sample sizes. The conclusions must be drawn appropriately based on the data presented.

Reviewer #2: Yes

Authors Reply: Not applicable

3. Has the statistical analysis been performed appropriately and rigorously?

Reviewer #2: Yes

Authors Reply: Not applicable

4. Have the authors made all data underlying the findings in their manuscript fully available?

The [PLOS Data policy](http://www.plosone.org/static/policies.action#sharing) requires authors to make all data underlying the findings described in their manuscript fully available without restriction, with rare exception (please refer to the Data Availability Statement in the manuscript PDF file). The data should be provided as part of the manuscript or its supporting information, or deposited to a public repository. For example, in addition to summary statistics, the data points behind means, medians and variance measures should be available. If there are restrictions on publicly sharing data—e.g. participant privacy or use of data from a third party—those must be specified.

Reviewer #2: No

Authors Reply: Made available

5. Is the manuscript presented in an intelligible fashion and written in standard English?

PLOS ONE does not copyedit accepted manuscripts, so the language in submitted articles must be clear, correct, and unambiguous. Any typographical or grammatical errors should be corrected at revision, so please note any specific errors here.

Reviewer #2: Yes

Authors Reply: Not applicable

6. Review Comments to the Author

Please use the space provided to explain your answers to the questions above. You may also include additional comments for the author, including concerns about dual publication, research ethics, or publication ethics. (Please upload your review as an attachment if it exceeds 20,000 characters)

Reviewer #2: My suggestions are basically improvements in English usage. I use my usual method, cut and paste, rather than tags in a pdf.
I think you should reconsider your answer to “data made available”. I think that means that your raw data (suitably anonymized) could be provided to another researcher who might want to check your analyses. You have asserted it is available because you showed the results in your tables, which misses the point. I defer to the editors to explain what they mean by “data made available”.

Authors Reply: anonymized data submitted

The figure at the end is so simple, it seems unnecessary. Yes, there are two numbers, and they are different from each other. That could be adequately conveyed with a sentence in the text. But if the editor is happy with it, let it be.
In “medical schools in Munich showed 23 % lifetime prevalence” and at least one other place, remove the space between the number and the % sign.
Authors Reply: Rectified
“was introduced in 2002 AD”. We know it is AD, common era (CE). Don’t need to say it.
“health workers

Authors Reply: Rectified
59 should be vaccinated timely.” Should be “health workers should be vaccinated in a timely manner” in order to be fully grammatically correct.
“There is no replacement to vaccines for effective protection.” Perhaps “no alternative” rather than “replacement”.

Authors Reply: Rectified
P 6. Change “Selected questions in knowledge section” to “Selected questions in the knowledge section”

Authors Reply: Rectified
Change “Knowledge related to Hepatitis B was assessed under five questions with some specific questions giving possible score ranged from 15-75” to “Knowledge related to Hepatitis B was assessed under five questions with some addition subquestions, giving a possible score range from 15-75”. I think that is what you mean. Maybe there were five questions but with 1a, 1b, … 5b, 5c or something.

Authors Reply: Rectified
Change “while questions for attitude and practice section were five making possible score range from 5-25” to “while there were five questions for the attitude and practice sections, making possible score ranges from 5-25”.

Authors Reply: Rectified
P 7. Change “to check the correlation between total score among knowledge and attitude, knowledge and practice, and attitude and practice” to “to check the correlation between total scores of knowledge and attitude, knowledge and practice, and attitude and practice”.

Authors Reply: Rectified

P 16. Change “The studies from Saudi Arabia and Ethiopia showed 86.5% and 84.6% respectively responded that vaccine prevents Hepatitis B” to “The studies from Saudi Arabia and Ethiopia showed 86.5% and 84.6%, respectively, responding that vaccine prevents Hepatitis B”.

Authors Reply: Rectified
P 18. Change “recall bias, information bias and, social desirability bias might have also affected” to ““recall bias, information bias, and social desirability bias might have also affected”. (move the comma)
Authors Reply: Rectified
P 19. I think “consent for publication” means that all the authors agree that it can be published, with their name on it; but maybe not.
Acknowledgements – was there no one who helped with the study, typing things up, or entering data, or facilitating access? Minor things not meriting authorship.

Authors Reply: All work related to this manuscript done by research team themselves
